# Social representation of masculine and feminine sports among Saudi adolescents

**DOI:** 10.3389/fpsyg.2024.1337157

**Published:** 2024-02-26

**Authors:** Munirah Alsamih

**Affiliations:** Department of Psychology, King Saud University, Riyadh, Saudi Arabia

**Keywords:** gender, social representations, sport, adolescents, Saudi Arabia

## Abstract

**Introduction:**

This study examined how certain sports are represented as masculine or feminine in Saudi adolescents, namely, which sports adolescents associate with males and which sports they associate with females. Previous research aligned with this concern was conducted within Western culture; however, there is a need to shed light on how the issues of social representation of masculine and feminine sports affect other cultures such as Middle Eastern cultures.

**Methods:**

A survey was completed by 280 Saudi adolescents, aged between 12 and 17 (*M* = 13.5, *SD* = 1.3). The survey contained open-ended recall questions that asked participants to name three masculine, feminine, and natural sports.

**Results:**

Most participants were familiar with using feminine and masculine terminology to describe sports, and nearly half had personally used gendered terms to describe sports. Overall, the participants generated 2,195 names of various sports, with the majority classified as natural (appropriate for both men and women), many masculine, and the fewest feminine.

**Discussion:**

The connection between specific sports and masculinity or femininity can restrict the activities of adolescents who do not conform to traditional gender roles. Also, adolescents who are interested in sports that are not typically associated with their gender may experience social stigma or exclusion, which can discourage their participation. Therefore, it is important to establish inclusive environments in sports, regardless of the gender.

## Introduction

Gender segregation in sports has created distinct and gendered views of certain activities. Sports can be divided into two categories based on characteristics which are locally perceived as either masculine or feminine: sports that are flexible and light are related to females. For example, football and boxing are typically considered masculine while sports such as gymnastics, figure skating, volleyball, cheerleading, and ballet are typically considered feminine. Finally, sports like tennis, basketball, swimming, and running are considered neutral and can be played by both genders (Riemer and Visio, 2003; Hardin and Greer, 2009; Chalabaev et al., 2013; Gentile et al., 2018; Sobal and Milgrim, 2019).

However, it has been recognized that sports do not have inherent gender characteristics; instead, societies impose gender roles and expectations on people from a young age, even in sports (Birrell, 2000). Riemer and Visio (2003) asked participants aged 4–19 years to rate a list of sports as either feminine, masculine, or neutral. The results showed that participants typically perceived football and wrestling as appropriate for males while aerobics and gymnastics were seen as appropriate for females. This supports traditional gender stereotypes. Adolescent girls in a focus group conducted by Slater and Tiggemann (2010) stated that one of the reasons for them to stop participating in sports is their desire to respect social conventions by not participating in masculine sports. In the most recent work by Cárcamo et al. (2021), boys and girls considered football a masculine sport while skating and volleyball were considered feminine sports.

In fact, children learn to categorize sports as masculine or feminine based on cultural norms; these categorizations guide their attitudes and behaviors about these sports. Social Representation Theory (SRT) (Farr and Moscovici, 1984) suggests that a society’s cultural and historical context shapes beliefs, attitudes and values. These constantly evolve through discourse and interaction with others. Individuals use socially shared representations, or “social representations,” to make sense of complex social phenomena and communicate with others.

SRT can be applied to gender and sport to illuminate how gender roles and beliefs about sports are constructed and reinforced through social interactions and media representations. For example, gender stereotypes in sports are perpetuated through media portrayals of male athletes as strong, aggressive, and dominant, while female athletes are portrayed as emotional, graceful, and less competitive (Kane et al., 2013; LaVoi et al., 2019). These representations influence perceptions of gender and sport and perpetuate gender stereotypes in sports participation and performance. Cultural contexts play a crucial role in shaping individuals’ attitudes toward sports (Xu et al., 2021). Although this topic has been extensively studied in Western culture, it is essential to shift attention to non-Western cultures and examine how social representations influence individuals’ attitudes from young age. A systematic review of physical inactivity, gender, and culture in Arab countries, including Saudi Arabia (Sharara et al., 2018), found that the participation of women in sports and physical activities is less than men due to adherence to social traditions.

In Saudi Arabia, gender roles are traditionally rigid and divided due to the influence of conservative Islamic teachings and cultural norms. This is reflected in various aspects of life, like sports. However, there has been a recent change in Saudi women’s participation in different areas of society, including sports. According to statistics from the Saudi Ministry of Sports (2020), there has been a 150% increase in Saudi women’s participation in sports in the last 5 years. The first Saudi woman participated in the Olympic Games in London in 2012. Saudi Arabia sent two female athletes: one in judo and one 800 m runner (Boykoff and Yasuoka, 2015). As part of Saudi Arabia’s Vision 2030 plan (Human Resources and Social Development, 2023), the government has recognized the importance of empowering woman and increasing women’s participation in various sectors. Thus, in recent years, the Saudi government has taken several steps to promote women’s sports and encourage female participation in physical activities. For example, the country has hosted international women’s sporting events, such as the Saudi Ladies International Golf Tournament, the Women’s International Friendly Tournament, and the Dakar Rally. The government has also established sports clubs and facilities exclusively for women and lifted some restrictions on women’s participation in sports; for example, they are allowed to attend football matches in stadiums (Alolian, 2022; Bose, 2023).

The goal of this study was to investigate how Saudi adolescents perceive certain sports as either masculine or feminine. Although, Sobal and Milgrim (2019) examined social representations of masculine, feminine, and neither-gendered sports, it had been limited to a Western cultural context and among university students specifically. So, this study seeks to expand the scope of the current literature for a more complete view on the social representation of gender typing in sports by investigating understudied cultures and populations. This study provides important insights into Saudi Arabia, a Middle Eastern culture, where societal norms strongly influence gender roles regarding physical activity and sport (Sharara et al., 2018). Previous studies that were conducted in Saudi Arabia have focused on the difference between males and females in physical activity and sports participation (e.g., Aljehani et al., 2022; Alharbi et al., 2024). Thus, there is a need to study such issues from a different perspective, and this study tries to solve that by shedding light on how particular sports consider as either masculine or feminine. Given the great effort by the Saudi government for social change, equality, and social inclusion in the field of sports (Boykoff and Yasuoka, 2015; Alolian, 2022; Bose, 2023), it would be important to examine how individuals perceived sports as masculine or feminine especially the young generation of adolescents. Indeed, adolescents are a vital population to study because they are in the process of developing their own gender identities and learning societal expectations about gender roles (Steensma et al., 2013).

## Methods

### Participants

This study included 280 Saudi adolescents, 130 girls and 150 boys, aged between 12 and 17 (*M* = 13.5, *SD* = 1.3).

The participants were recruited using two ways: direct contact with schools and snowball sampling techniques. To recruit participants from schools, the researcher contacted the education ministry to provide a letter for schools to facilitate the recruiting. After obtaining the letter, around 10 elementary and secondary public schools in the center region of Saudi Arabia were reached with the details of the study after the school administration showed a willingness to collaborate.

### Procedures

Before data collection, ethical approval was obtained from the Institutional Review Board at (anonyms). School administration distributed the study details along with the parental consent form to students. Students who returned the consent form, signed by their parents, were provided with a barcode to access the electronic questionnaire and fill it out at home. Additionally, the research author reached out to some mothers who had previously participated in studies with their children and explained the research to them. The author requested that they refer their friends who would be willing to have their children participate. Subsequently, a link to the questionnaire was sent to the parents to pass on to their children.

After data collection, the surveys were checked for completeness and accuracy. The answers to open-ended questions were coded and entered into SPSS for analysis along with the quantitative data. Descriptive statistics and inferential statistics were calculated to analyze the data.

### Measures

A questionnaire was developed based on the free association task, often used to study social representations. This involves participants being asked to recall and list a certain number of words or names that come to mind when they think of a specific topic or concept. This helps researchers to understand the structure and content of the social representations associated with that topic (Idoiaga et al., 2020; Martikainen and Sakki, 2021).

The questionnaire was adapted from Sobal and Milgrim’s (2019). It consisted of five questions (Appendix 1). The first two questions were yes or no questions that measured the participants’ awareness and use of social representations related to gender stereotyping in sport. The first question was, “Have you ever heard sports referred to as ‘feminine’ or ‘masculine’?” The second question was, “Have you ever personally referred to sports as ‘feminine’ or ‘masculine’?” Then, participants were asked to name three sports that they considered feminine, three that they considered masculine and three that they considered both genders.

The questionnaire was pilot tested on 12 adolescents (11 girls and a boy) a similar to those who would participate in the final survey to ensure its clarity and understandability; no significant changes were made.

### Data analysis

The first two questions were scored as 0 for “yes” and 1 for “no.” The counts and percentages for each response were calculated to determine adolescents’ awareness and use of social representations related to gender stereotyping.

For the open-ended questions, where participants were asked to name three sports that they considered feminine or masculine for both women and men, the author coded and compiled responses. A numerical code was assigned to each sport to facilitate data entry into SPSS. The outcomes were presented in terms of frequencies and proportions, with the prevailing responses exemplifying the central social representations, while the uncommon responses signified marginal or secondary social representations (Abic, 1993; Sobal and Milgrim, 2019).

## Results

### The presence and use of gender-stereotyped sports terminology

Overall, 79% of the participants stated they were familiar with using feminine or masculine terminology to describe sports (48% of girls and 52% of boys). In addition, 47% of the participants had personally used gendered terms to describe sports (49% of girls and 51% of boys). In general, the results for boys and girls were relatively similar.

### Social representations of gender-stereotyped sports

For feminine sports, 88% named three sports, 5% reported two sports, 5% reported only one female sport, and 15% reported no sports. For masculine sports, 88% named three sports, 4% reported two male sports, 3% reported one male sport, and 10% named no sports. For neutral sports, 94% named three sports, 6% reported two sports, 5% reported only one sport, and 6% reported no sports. Overall, the participants generated 2,195 names for various sports, with 737 classified as masculine, 698 as feminine, and 760 as neither masculine nor feminine (Table 1). The named sports fell into 62 distinct categories across the three gender classifications.

**Table 1:** Feminine, masculine and both genders sports named by adolescents.

	Feminine sports (*N* = 698)		Masculine sports (*N* = 737)		Both genders (*N* = 760)	
Sport	*n*	%	*n*	%	*n*	%
Swimming	65	23.6%	28	10.0%	132	45.5%
Horse riding	16	5.8%	10	3.6%	35	11.8%
Climbing	3	1.1%	4	1.4%	6	2.0%
Volleyball	38	13.8%	49	17.5%	63	23.1%
Basketball	54	19.6%	118	42.1%	78	27.5%
Running	28	10.1%	15	5.4%	42	15.7%
Football	56	20.3%	183	65.4%	118	42.4%
Cycling	5	1.8%	8	2.9%	16	5.9%
Gymnastics	67	24.3%	1	0.4%	9	2.4%
Tennis	66	23.9%	22	7.9%	62	23.5%
Ballet	66	23.9%	0	0.0%	1	0.4%
Badminton	6	2.2%	0	0.0%	2	0.4%
dance	32	11.6%	0	0.0%	2	0.8%
Baseball	3	1.1%	10	3.6%	4	1.2%
Ground tennis	1	0.4%	2	0.7%	2	0.4%
Karate	1	0.4%	14	5.0%	7	2.7%
Tae Kwon Do	0	0.0%	4	1.4%	4	1.2%
Skating	7	2.5%	2	0.7%	3	1.2%
Rowing	1	0.4%	2	0.7%	0	0.0%
Handball	8	2.9%	9	3.2%	8	3.1%
Yoga	38	13.8%	0	0.0%	6	2.0%
Walking	14	5.1%	2	0.7%	37	12.5%
Squash	2	0.7%	0	0.0%	0	0
Hockey	5	1.8%	6	2.1%	8	2.7%
Badel	3	1.1%	1	0.4%	4	1.2%
Table tennis	4	1.4%	2	0.7%	5	1.6%
Zumba	7	2.5%	0	0.0%	0	0.0%
Bowling	5	1.8%	2	0.7%	6	2.4%
Kickboxing	2	0.7%	4	1.4%	0	0.0%
Ice skating	8	2.9%	0	0.0%	3	0.8%
Surfing	2	0.7%	1	0.4%	2	0.4%
Weight-lifting	6	2.2%	53	18.9%	10	3.1%
Modern dance	2	0.7%	0	0.0%	0	0.0%
Golf	10	3.6%	10	3.6%	20	7.1%
Fencing	0	0.0%	0	0.0%	2	0.8%
Softball	2	0.7%	1	0.4%	0	0.0%
Boxing	1	0.4%	56	20.0%	11	3.5%
Cycling race	3	1.1%	5	1.8%	0	0.0%
Martial arts	3	1.1%	3	1.1%	1	0.4%
shooting	3	1.1%	4	1.4%	7	2.0%
American football	1	0.4%	15	5.4%	0	0.0%
Javelin	0	0.0%	1	0.4%	0	0.0%
Wrestling	1	0.4%	22	7.9%	0	0.0%
Tent pegging	0	0.0%	1	0.4%	0	0.0%
Camel racing	0	0.0%	1	0.4%	0	0.0%
Rugby	0	0.0%	2	0.7%	0	0.0%
Car racing	0	0.0%	4	1.4%	0	0.0%
Kung fu	0	0.0%	4	1.4%	0	1.6%
Bullfighting	0	0.0%	1	0.4%	0	0.0%
Show jumping	0	0.0%	2	0.7%	1	0.0%
Motorcycle racing	0	0.0%	2	0.7%	0	0.4%
Judo	1	0.4%	2	0.7%	0	0.0%
Hammer throw	0	0.0%	1	0.4%	0	0.0%
High jump	0	0.0%	2	0.7%	0	0.0%
Sprint	1	0.4%	3	1.1%	1	0.4%
Discus throw	0	0.0%	1	0.4%	1	0.4%
Chess	1	0.4%	0	0.0%	1	0.4%
Polo	0	0.0%	0	0.0%	1	0.4%
Marathon	0	0.0%	0	0.0%	5	2.0%
Camel riding	0	0.0%	1	0.4%	0	0.0%
Self defense	2	0.7%	1	0.4%	1	0.4%
Meditation	0	0.0%	1	0.4%	1	0.0%

The most frequently named feminine sports were gymnastics (24.3% of all feminine responses), tennis (24%), ballet (24%), swimming (24%), basketball (20%), football (20%), volleyball (14%), yoga (14%), and running (10%). These sports represent 67% of all sports classed as feminine. Overall, 54 sports were classed as feminine by at least one participant. Notably, participants named three types of dance: ballet, modern dance, and Zumba.

The most frequently named masculine sports were football (65% of all masculine responses), basketball (42%), boxing (20%), weightlifting (19%), volleyball (18%), and swimming (10%). These include 54% of all sports classed as masculine. Overall, 61 sports were named masculine sports by at least one participant.

The most frequently cited neutral sports were swimming (47% of all neutral responses), football (42%), basketball (28%), tennis (22%), and walking (13%). These constitute 65% of all sports classed as natural. Overall, 52 sports were recalled as neutral by at least one participant.

## Discussion

This study investigated how Saudi adolescents perceive certain sports as either masculine or feminine. Most participants were familiar with using feminine and masculine terminology to describe sports, and nearly half had personally used gendered terms to describe sports. These results are consistent with prior research on gender and sports, showing that gendered language is often used to describe sports and that gender stereotypes are deeply ingrained in societies (Sobal and Milgrim, 2019).

The use of gendered language around sports may reflect broader cultural beliefs and values around gender and physical activity, highlighting their importance as social representations (Abic, 1993). In many cultures, including Saudi Arabia, masculinity is strongly associated with physical strength, while femininity is associated with fragility and weakness. This can lead to a gendered division of labor and leisure activities, with men typically engaging in more physically demanding and competitive sports, while women are encouraged to participate in more passive or domestic activities (Gentile et al., 2018; Zipp and Nauright, 2018; Alruwaili, 2020).

Social representations theory (Farr and Moscovici, 1984) suggests that the use of gendered language and social representations of sports is particularly salient and influential among adolescents because they are still developing their beliefs, attitudes, and behaviors around gender and physical activity. They may be more susceptible to the influence of societal and cultural norms because they are undergoing socialization and learning how to navigate social expectations and roles.

The social representations of masculine, feminine, and non-gendered sports in this study are consistent with previous research conducted by Sobal and Milgrim (2019) that recognized masculine sports as crucial social representations, feminine sports are considered less important social representations, while non-gendered sports show more gender inclusivity in sport.

Social representations of feminine sports showed that gymnastics, tennis, ballet, swimming, basketball, football, volleyball, yoga, and running were the most frequently named feminine sports among Saudi adolescents in this study. Saudi Arabia has strict gender norms; traditionally, physical activities have been restricted for women and girls. However, recent significant efforts have been made to promote sports participation among women and girls in the country (Boykoff and Yasuoka, 2015; Bose, 2023). The diverse range of sports that the adolescents cited as feminine may indicate a change in the social representations of sports among the younger generation in Saudi Arabia. Notably, ballet and gymnastics were ranked as the top two feminine sports. This suggests that adolescents in Saudi Arabia view these sports as particularly feminine. Research has shown that gymnastics and ballet are associated with femininity in many cultures worldwide (Riemer and Visio, 2003; Hardin and Greer, 2009; Sobal and Milgrim, 2019). Indeed, the social representation of gymnastics and ballet as a feminine sport is not unique to Saudi Arabia but is a widely recognized cultural phenomenon. The reasons for this social representation may vary across cultures, but it is likely influenced by factors such as social norms, gender role expectations, aesthetics, and sporting performance.

Overall, the results highlight that some sports were socially represented as feminine among Saudi adolescents. These findings could inform efforts to promote sports participation among girls and women in Saudi Arabia by indicating the sports where women will face participation challenges and the barriers that must be addressed to combat these.

Reflecting previous research (Riemer and Visio, 2003; Hardin and Greer, 2009; Sobal and Milgrim, 2019), football, basketball, and boxing were the most commonly named masculine sports by Saudi adolescents. Football was by far the most frequently named masculine sport. This finding indicates a strong cultural association between football and masculinity in Saudi Arabia. From the perspective of social representations (Farr and Moscovici, 1984), football in many cultures is represented as a hegemonic masculine sport (Hardin and Greer, 2009; Sobal and Milgrim, 2019).

Notably, volleyball and swimming were also mentioned as masculine sports, despite being among the top sports associated with femininity. This may indicate that the social representations of certain sports may differ depending on the gender of the participant or the context in which they are played.

Weightlifting and boxing were represented as masculine sports. This suggests that Saudi adolescents may view strength and physical prowess as qualities associated with masculinity. This is consistent with previous research (e.g., Sobal and Milgrim, 2019) identifying that physical strength and athleticism are associated with masculinity in many cultures worldwide.

Swimming was the most frequently named neutral sport, suggesting it is a popular and accessible sport in Saudi Arabia. Indeed, some Saudi families have a swimming pool in their houses, and some families rent a chalet with a swimming pool during weekends and vacations. This may explain why swimming was considered a sport for both genders. Tennis and walking were also among the most commonly cited neutral sports. This suggests a growing interest in health and fitness among the Saudi youth, with a greater focus on individual sports and activities that promote physical well-being, such as walking. The Saudi Ministry of Health and the Saudi Federation of Sports for All have collaborated to launch several campaigns encouraging both genders and different ages to walk more (e.g., the Walk 30 campaign) (Saudi Ministry of Health, 2020).

Overall, the results suggest that the social representations of certain sports as neutral in Saudi Arabia, reflecting broader cultural norms and expectations about sports participation. The findings also support that there is a growing interest in individualistic and fitness-oriented activities in Saudi Arabia, which may reflect broader trends toward healthier lifestyles and an increased focus on personal well-being.

There are also efforts to promote sports participation among women and girls in Saudi Arabia (Bose, 2023). These efforts may be reflected in the significant overlap between feminine and masculine sports in this study’s findings. This overlap may also reflect changing attitudes about gender roles and sports participation. As more girls and women become involved in sports, there may be increased recognition of individuals’ diversity of interests and abilities, regardless of gender. Additionally, the new generation has become more exposed to different cultures through social media, which may lead them to perceive greater gender equality in many sports. Many participants had probably watched or heard of the Olympics; this may have provided them with insight into sports that both genders can play.

## Limitations and future research

While 280 participants is a decent sample size, it may not represent all Saudi adolescents. This limits this research’s generalizability; further research with more extensive and more diverse samples should the study’s findings. Furthermore, the results may not be generalizable to other cultures with different gender norms and expectations, considering the strict gender norms and expectations that exist in Saudi Arabia.

For future research, it would be beneficial to conduct longitudinal studies that investigate how adolescents’ perceptions of gender stereotyping in sports change over time. Additionally, conducting qualitative studies could provide a more comprehensive understanding of how masculine and feminine sports are socially represented. This could involve interviews or focus groups to explore the reasons behind adolescents’ responses and the factors influencing their perceptions. Also, comparing Saudi Arabia with different cultures could illuminate the extent to which cultural factors influence gender and sports perceptions. Finally, intervention studies to promote gender equity in sports among adolescents could highlight effective strategies for challenging gender stereotypes and promoting inclusivity in sports.

## Conclusion and implications

This study has highlighted the need for continued research and dialogue about gender and sports in Saudi Arabia and other cultural contexts. Examining the social and cultural factors that shape the formation and expression of gendered language and social representations of sports helps researchers and practitioners to develop more nuanced and effective strategies for promoting gender equity and inclusivity in sports and physical activity. For example, initiatives that challenge traditional gender roles and stereotypes, promote girls’ and women’s participation in sports, and encourage gender-neutral language around sports may help to create a more inclusive and equitable sports culture in Saudi Arabia and beyond.

As Saudi Arabia undergoes social and cultural changes, the division of gender roles in sports may become less distinct, potentially leading to a decrease in gendered terminology use. However, this claim is speculative and further research should be conducted to confirm it.

Furthermore, the association between certain sports and masculinity or femininity can be limiting for adolescents who do not conform to traditional gender norms. Adolescents interested in sports that are not typically associated with their gender may face social stigma or exclusion, which may discourage their participation. Therefore, it is vital to create inclusive environments for all adolescents to participate in sports and physical activities, regardless of gender identity or expression.

## Data availability statement

The raw data supporting the conclusions of this article will be made available by the authors, without undue reservation.

## Ethics statement

The studies involving humans were approved by Institutional Review Board in King Saud University. The studies were conducted in accordance with the local legislation and institutional requirements. Written informed consent for participation in this study was provided by the participants’ legal guardians/next of kin.

## Author contributions

MA: Writing – original draft, Writing – review & editing.

## Appendix 1

*Questionnaire*

1. Have you ever heard sports referred to as ‘feminine’ or ‘masculine’?

- -Yes -No

1. Have you ever personally referred to sports as ‘feminine’ or ‘masculine’?

- -Yes -No

1. Name three sports that you considered “feminine”
2. Name three sports that you considered masculine
3. Name three sports that you considered for both genders
